# Porcine Epidemic Diarrhea Virus Variants with High Pathogenicity, China

**DOI:** 10.3201/eid1912.121088

**Published:** 2013-12

**Authors:** Jinbao Wang, Pengwei Zhao, Lihui Guo, Yueyue Liu, Yijun Du, Sufang Ren, Jun Li, Yuyu Zhang, Yufeng Fan, Baohua Huang, Sidang Liu, Jiaqiang Wu

**Affiliations:** Shandong Academy of Agricultural Sciences, Jinan, China (J. Wang, L. Guo, Y. Du, S. Ren, J. Li, Y. Zhang, Y. Fan, B. Huang, J. Wu);; Shandong Key Laboratory of Animal Disease Control and Breeding, Jinan (J. Wang, P. Zhao, L. Guo, Y. Du, S. Ren, J. Li, Y. Zhang, B. Huang, J. Wu);; Shandong Agricultural University, Tai-an, China (J. Wang, Y. Liu, S. Liu)

**Keywords:** Porcine epidemic diarrhea virus, variants, animal infection experiment, viruses, China, pigs

**To the Editor:** Porcine epidemic diarrhea (PED), a serious and highly contagious swine disease, is characterized by severe diarrhea and dehydration in suckling piglets. The etiologic agent, porcine epidemic diarrhea virus (PEDV), is an enveloped, single-stranded RNA virus (family *Coronaviridae*, order Nidovirales). The viral disease was discovered in the United Kingdom in 1971 and subsequently reported in many swine-producing countries in Europe and Asia (*1*,*2*). Although most sow herds previously had received CV777-based inactivated vaccine, a large-scale outbreak of PED has been associated with high rates of illness and death in suckling piglets in China since late 2010, resulting in substantial economic losses.

We collected 217 piglets (126 alive, 91 dead) with diarrhea from 42 farms in Shandong Province, China, during November 2010–April 2012. To determine the etiologic agent of the outbreak, we analyzed samples of intestine and its contents. A total of 175 (80.6%) samples were PEDV positive, indicating that PEDV was the dominant pathogen for this diarrheal outbreak. Other pathogens also were identified: porcine transmissible gastroenteritis virus (8.3%), rotavirus (3.7%), and *Escherichia coli* (3.2%). Furthermore, 8.1% of pigs with diarrhea were co-infected by 2 pathogens.

Three PEDV field strains (ZB, YS, and SH) were isolated from different farms where 100% of sucking piglets had diarrhea. The virus isolates were plaque-purified once in Vero cells. The spike protein (S), encoded by S gene of PEDV, plays a pivotal role in cell adsorption, membrane fusion, and induction of neutralizing antibodies (*3*,*4*). The full-length S genes of 3 isolates were amplified by reverse transcription PCR with 2 pairs of primers (*5*) and sequenced to identify the genetic variation of the isolates.

Sequences for the S gene were submitted to GenBank (accession nos. for YS: JQ771753; SH: JQ771751; and ZB: JQ771752). The S gene nucleotide sequences and deduced amino acid sequences of the 3 new isolates were aligned with the sequences of published isolates by using MEGA 4.0 (www.megasoftware.net). S protein identity among the 3 new isolates was 99.4%–99.6% and shared 93.6%–93.7% identity with classical CV777 strain. We identified numerous sequence variations in S protein of the 3 isolates (Technical Appendix Table). Two separate insertions were discovered: a 1-aa (N) insertion at position 140 and a 4-aa (QGVN) insertion at positions 59–62. A 2-aa (NI) deletion was identified at positions 163–164. A total of 34 separate substitutions were identified, and the number(s) of replaced amino acids ranged from 1 through 5. These sequence variations were similar to those in CH/FJND-3/2011, CH1, CH8, and CHGD-01 isolates recently reported in China (*6*).

To determine the virulence of the PEDV isolates, we experimentally infected fifteen 4-day-old Duroc crossbred piglets with the YS and ZB isolates. The piglets were randomly allotted to 3 groups, each group consisting of 5 pigs. These piglets were shown by serologic analysis to be negative for antibodies against PEDV, porcine reproductive and respiratory syndrome virus, porcine transmissible gastroenteritis virus, and pseudorabies virus.

In the piglets inoculated orally with 1.5 mL of YS isolate (10^3.0^ 50% tissue culture infectious doses/mL), diarrhea was observed at 3–6 days postinfection (dpi). One piglet died at 5 dpi, and 4 piglets died at 6 dpi. The dead piglets showed hemorrhage and shedding in the gastric mucosa, swelling and congestion in the mesenteric lymph nodes, and hemorrhage in the intestinal wall (Figure, panel A). Histopathologic changes included epithelial cell shedding; intrinsic layer hemorrhage and excessive infiltration of lymphocytes in the stomach; and congestion, edema, and epithelial cell shedding in the intestinal mucosa (Figure, panel B). PEDV was recovered from the dead piglets, and the full-length S gene was amplified by reverse transcription PCR and sequenced. In the S genes of YS and ZB isolates, 3 and 2 single nucleotides were replaced, respectively, but no mutated amino acid was introduced between the inoculated and recovered viruses.

**Figure:**
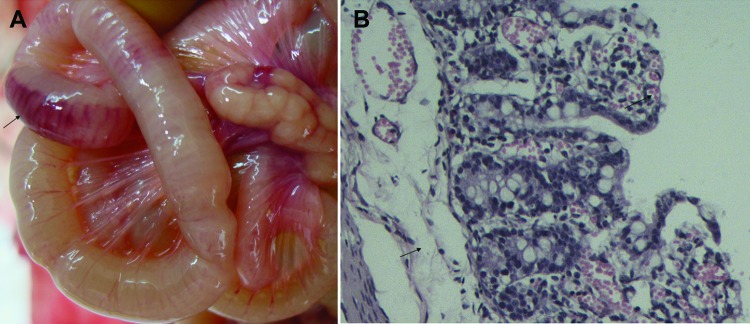
Signs of porcine epidemic diarrhea virus in piglets, China, November 2010–April 2012. A) Hemorrhage in the intestinal wall. B) Congestion, edema, and epithelial cell shedding in the intestinal mucosa. Hematoxylin and eosin stain; original magnification ×200.

For the piglets orally infected with 1.5 mL of ZB isolate (10^3.1^ 50% tissue culture infectious doses/mL), diarrhea was observed at 3–5 dpi, and all 5 piglets died at 5 dpi. The dead piglets showed similar lesions to those of the piglets infected with YS isolate in the stomach, intestines, and mesenteric lymph nodes. Piglets in the control group orally inoculated with Dulbecco minimal essential medium remained healthy during the experiment, and no obvious pathologic changes were observed.

Our investigation indicated that the recent diarrhea outbreaks were mainly caused by PEDV variants with novel genetic markers that distinguish them from classical strains. The YS and ZB isolates were highly virulent in piglets. Unlike CV777, the PEDV variants remained almost unchanged in the epitope at positions 499–638; however, a 2-aa deletion, a 1-aa insertion, and 18 separate substitutions were identified in the epitope at positions 83–276 (*7*,*8*). These variations of amino acid sequences probably changed the immunogenicity of S protein and led to immunization failure of current commercial vaccines made from classical PEDV strains. However, how PEDV has evolved and varies in pig herds are not clear. Further studies, including extensive genomic sequence analyses and serologic cross-neutralization tests, should be conducted.

Technical Appendix Summary of amino acid mutations in S protein of 3 new isolates of porcine epidemic diarrhea virus, China.
