# Supplementary material for: Porcine Epidemic Diarrhea Virus Variants with High Pathogenicity, China
Source: Emerg Infect Dis. 2013 Dec;19(12):2048–9. doi: 10.3201/eid1912.121088 (PMC3840889; doi:10.3201/eid1912.121088)
Supplement: Technical Appendix — Summary of amino acid mutations in S protein of 3 new isolates of porcine epidemic diarrhea virus, China. [file 12-1088-Techapp-s1.pdf]

Article DOI: http://dx.doi.org/10.3201/eid1912.121088

Porcine Epidemic Diarrhea Virus Variants with High Pathogenicity, China

Technical Appendix

Technical Appendix Table. Summary of amino acid mutations in S protein of 3 new isolates of porcine epidemic diarrhea virus, China, November 2010–April 2012

Mutations	Amino acid nos.	Amino acid changes	Location in amino acid
Substitutions	3	QST→SY (A) N	27–29
	4	SMNS→IGEN	55–58
	1	S→T	64
	5	GTGIE→AGQHP	68–72
	1	L→V	82
	1	Y→H	84
	1	D→R	86
	1	S→G	87
	1	Q→H	89
	1	I→T	120
	2	DN→SI	130–131
	1	V→A	138
	4	QDGK→SEHS	159–162
	1	A→S	179
	1	H→Y	184
	1	L→F	187
	1	R→K	197
	3	KRS→SGG	201–203
	1	T→E	211
	1	Y→S	228
	1	E→Q	230
	2	DS→EP	247–248
	1	L→V	271
	1	M→I	305
	1	L→F	347
	1	N→S	724
	1	A→V	964
	1	S→A	1049
	1	N→D	1167
	1	G→D	1178
	2	TY→NH	1198–1199
	1	R→Q	1303
	1	G→C	1364
	1	A→V	1381
Insertions	4	QGVN	59–62
	1	N	140
Deletions	2	NI	163–164
